# Gleanings from Our Exchanges

**Published:** 1872-12-15

**Authors:** 


### Gleanings from Our Exchanges.

Treatment of Scarlet Fever.—The late Prof. Geo. T. Elliott, in a lecture on this disease, gave the following method of treatment: To bring the eruption out, if it has not already presented itself, order hot baths and blankets. Give nothing to eat at first in the eruptive stage, and only allow the simplest nourishment the first day. Patients experience great relief from baths, and the application of cold cream, or mutton tallow, over the whole body. Visit the patient twice a day. By pouring a pitcher full of cold water over the back of the neck, especially when the glands are enlarged, great comfort is experienced. As a gargle, make use of chlorate of potash or soda. Pieces of ice are good, in the mouth. Sprays, thrown in with Richardson's instrument, of lime water, solutions of alum and sulphate of zinc, are beneficial. As a palative to the throat, the vapor from slaked lime can be recommended. Strong beef tea, with opium may be thrown up the bowel. Begin to feed the patient from the second day of the eruption with animal essences. If the tonsils are enlarging and the pharynx exhibits much redness, with diphthertic exudation, the physician has a right to say that things look bad. If the throat symptoms do not mitigate on the fourth or fifth day, the voice being affected, then one feels that there is a good deal of danger. When the kidneys show hyperæmia, desquamation, or transitory albuminuria, then there is a twofold danger. Always examine the urine. When the patient has kidney disease, the treatment should be directed to the skin and bowels; when the latter are loaded and constipated, give powerful saline cathartics. Get Ronochetti's apparatus, to produce perspiration. To convalescing patients the use of iron is beneficial. The bisulphites have been recommended, but, from experience, they cannot be advocated. Belladonna is not always a prophylactic, although, on account of its innocence, and a feeling of satisfaction to the practitioner and family, it is well to administer it.

Hæmorrhoidal Tumors of the Female Urethra.—The *Journal de Med. et de Chir. Pratique*, contains an article by M. Richet, on this affection, in which this gentleman calls attention to the fact that many of the painful tumors found in this locality are hæmorrhoidal, and not, as is sometimes supposed, polypoid growths from the mucous membrane. The galvanic cautery and forcible dilatation are spoken of as the most successful modes of treatment.—*Med. Record.*

Conservative Surgery in Minor Operations.—Dr. A. P. Grinnell says, in the New York *Medical Journal:* A lad, aged fourteen, of a vigorous, healthy constitution, received a severe wound on the right hand by a circular saw. Upon examination of the injury, I found that the saw had entirely severed the third finger, with the exception of a little cuticle, which held the end suspended between the first and second articulation, had disarticulated the first and second phalanges at the second joint, and, besides, injured the soft parts in different parts of the hand. My first thought was to finish the amputation of all dependent portions, but, upon reflection, I determined to afford nature an opportunity of showing her skill in establishing a union of these lacerated tissues. The subsequent treatment involved much care and attention, but the result was sufficiently gratifying to reward the effort of preserving the whole hand. We have been taught that, in all injuries of this character, where union has taken place, sensation was lost, and the power of motion considerably impaired; but, in the above case, bone united firmly to bone, tendon with tendon, nerve with nerve, and the circulation of blood was fully restored. Sensation is perfect beyond the point of injury; and, to have secured this result, nerve must have united with nerve, for the amputations of all sensitive portions was complete. The power of motion is also preserved, although not perfect in one finger, owing to anchylosis of a joint.

Vehicle for the Internal Administration of Chloroform (*Journal de Pharm. et de Chimie*, 1872).—Dr. G. W. Murdonck, having tried various formulæ proposed to facilitate the ingestion of chloroform, has found that some were difficult of execution, others contained sulphuric ether, and others again contained but little chloroform. He considers the best proceeding to consist in dissolving the chloroform in glycerin (1:3), which is effected with tolerable facility, and gives a very clear solution, pleasant to the taste, and with a strong odor of chloroform. This solution can be mixed in all proportions with water, without the occurrence of any precipitation, though the odor is distinctly perceptible. In forming the mixture it is well to add the chloroform slowly, and to mingle the two thoroughly. It should be left at rest for twenty-four hours; at the expiration of this period, a portion of the chloroform will be found to have collected at the bottom of the vase; this should be separated and mixed with an additional part of glycerin, when no further separation will occur. This mixture may be kept for some time without any loss of chloroform by evaporation.—*Med. Times.*

Ozone in the Atmosphere. — Some researches on native ozone are given in the *Journal de Pharmacie*, by M. A. Houzeau. The air in the open country, taken at a distance of 2 metres above the ground, contains at most, according to this author's investigations, 1-450,000 of its weight of ozone, or 1-100,000 part of its volume. (Density of ozone 1.658 according to Loret.) This amount increases the farther it is from the ground, though it is in all cases quite variable. Litmus paper half iodized in the atmosphere showed the following colors:

NUMBER OF HOURS EXPOSED IN CALM AIR.

| 2 | 4 | 6 | 8 | 10 | 12 | 24 |
|---|---|---|---|---|---|---|
| none | violet | very pale blue | very pale blue | blue | blue | very blue |

NUMBER OF HOURS EXPOSED IN AIR AGITATED.

| 2 | 4 | 6 | 8 | 10 | 12 | 24 |
|---|---|---|---|---|---|---|
| pale violet | violet | pale blue | blue | very blue | very blue | blue |

Its origin is undoubtedly electric, and this the author believes is due in great measure to the silent discharge of electricity from the clouds to the earth.—*Med. Reporter.*

The Phenomena of Menstruation.—The views of Pfluger (*Schroeder's Manual of Obstetrics*) with reference to the cause of the phenomena of menstruation are as follows: A constant irritation is exerted on the extremities of the nerves imbedded in the fibrous stroma of the ovary by the slow but uninterrupted growth of the Graafian follicle. This is not sufficiently intense to produce reflex action at once, but in the intermenstrual period the total irritation is so great that reflex action takes place in the form of marked arterial congestion. This sudden increase in the amount of blood produces essentially two results: In the first place, that Graafian follicle which is the farthest advanced in development ruptures, in consequence of the increased intra-follicular pressure; while, in the second place, a hæmorrhage takes place from the free surface of the uterine mucous membrane; hence the escape of the ovum from the follicle, and the menstrual flow, are joint effects of one and the same cause, namely, the pressure which the developing follicle exerts on the extremities of the nerves, which are distributed throughout the ovarian stroma.—*Gaz. Hebdom.*

Hydropneumothorax.—Sir Henry Thompson, M.D., F.R.C.S. (*British Medical Journal*, Oct. 5th, 1872) in a clinical lecture on this rare disease, stated that he had been induced to modify some of the opinions formerly expressed by him. He was of the opinion, whatever the view taken of the case on admission—whether the man was phthisical or not—whether a gap in the pleura existed at the time or not—under all circumstances, an operation was unavoidable. But the drainage-tubes ought to have been inserted either on the instant, or, at any rate, after the discovery of the following conditions: Free and direct communication between the left pleural cavity and the trachea, advancing disorganization of the right lung, and ever-increasing accumulation of pus, in spite of its enormous discharge. By adopting these measures, the pus might have been kept within bounds, and diverted from the trachea and bronchi; the pneumonic processes, which were so rapidly disabling the only lung of any real use in respiration, might have been arrested; and although the man must invariably have died an early death from combined exhaustion and apnœa, he said that the patient might have been saved from dying of downright suffocation—literally drowned in pus.—*Br. Med. Journal.*

Prof. Agassiz's Expedition.—At the welcome extended to Prof. Agassiz by the California Academy of Sciences, on his arrival at San Francisco with the Hassler Expedition, the speaker reported the following results of the Expedition:

During the 246 days of the voyage there were packed 242 boxes and barrels of specimens. The materials collected will oblige the trustees of the Museum at Cambridge, Mass., to build an extensive addition. The collection of fishes amounts to about 30,000 specimens. He has no doubt that the sum total of specimens brought together exceeds 100,000, and they are not preserved in the old-fashioned way—dried and unfit for further research. To pack these specimens, 3,000 and odd gallons of alcohol were used up, and they are so preserved they will afford materials for investigation for years to come.

Internal Pathology.—Don Jose Zalabardo, of Madrid (Siglo Medico) announces a work on "Internal Pathology," in verse, which will be abreast of the present state of science.

